# Establishing a national mortality review programme for people with intellectual disabilities: The experience in England

**DOI:** 10.1177/1744629520970365

**Published:** 2020-11-18

**Authors:** Pauline Heslop, Victoria Byrne, Rachel Calkin, Kamila Gielnik, Avon Huxor

**Affiliations:** 1980 University of Bristol, UK; 1980 University of Bristol, UK; 1980 University of Bristol, UK; 1980 University of Bristol, UK; 1980 University of Bristol, UK

**Keywords:** mortality review, LeDeR programme, intellectual disabilities, service improvement

## Abstract

In England, the national mortality review programme for people with intellectual disabilities, the LeDeR programme, was established in 2015. The programme supports local areas to review the deaths of all people with intellectual disabilities aged 4 years and over. Each death has an initial review; if indicated, a full multi-agency review takes place. The learning from the mortality reviews contributes to service improvements locally and nationally. This paper describes the programme’s introduction and processes, exploring the challenges faced, and the successes achieved. It considers the background and rationale for the programme and the steps taken during its implementation, in order that others can learn from our experiences. Now the programme is established, its focus needs to shift so that we have a better understanding about how the findings of mortality reviews are leading to local and national service improvements and their impact.

## Introduction

This paper describes the introduction and development of the Learning (Intellectual) Disabilities Mortality Review (LeDeR) programme in England.

The context within which the LeDeR programme was introduced was the need to reduce the apparently high rates of premature mortality in people with intellectual disabilities in England. Arguably, it was reports (e.g. Mencap, 2004, 2007; Michael, 2008; Heslop et al., 2013) and reviews (e.g. Mazars, 2015; Parliamentary and Health Service Ombudsman, 2009) that documented the disparity in age at death between people with intellectual disabilities and the general population, alongside campaigns driven by families of those who had died (e.g. Justice for LB – http://justiceforlb.org/), that were the key driving forces for the programme.

Statistical evidence about inequalities in mortality of people with intellectual disabilities had been accumulating in England (Glover et al., 2017; Hosking et al., 2016) and internationally (Arvio et al., 2016; Trollor et al., 2017). Most of these reports noted an approximate 20-year disparity in age at death for people with intellectual disabilities compared to the general population. In England, NHS Digital published experimental statistics introducing a 3-year pooled mortality indicator for people with intellectual disabilities; for the years 2015–2018 the standardised mortality ratio in England was 403 (Confidence Interval 393–417) (NHS Digital, 2019). In other words, people with intellectual disabilities aged 0 to 74 years were about four times more likely to die than would be expected for people without intellectual disabilities.

A large proportion of premature deaths of people with intellectual disabilities were considered avoidable through the provision of good quality health and social care (Hosking et al., 2016; Trollor et al., 2017; Heslop et al., 2013). Contributory factors to early deaths included:

- Professional ‘indifference’ or discriminatory attitudes e.g. healthcare professionals relying inappropriately on their own estimates of a person’s quality of life (Mencap, 2007; Michael, 2008).
- Health and social care professionals not listening to those who knew the person well when they voiced concerns about the person’s health (Mazars, 2015; Parliamentary and Health Service Ombudsman, 2009; Heslop et al., 2013).
- A lack of understanding and training about intellectual disabilities among health and social care professionals (Mencap, 2012; Michael, 2008).
- A lack of partnership working, communication and coordination across and between services (Mazars, 2015; Heslop et al., 2013).
- Insufficient attention given to making reasonable adjustments to support the delivery of equal treatment (Parliamentary and Health Service Ombudsman, 2009; Heslop et al., 2013).
- Difficulties accessing health care, delays in diagnosis, and poor management (Michael, 2008; Heslop et al., 2013).

Following the publication of the Confidential Inquiry into premature deaths of people with learning disabilities (CIPOLD) in 2013, and its findings from detailed reviews of the deaths of 247 people with intellectual disabilities in England, the (former) Department of Health (DH) in England reported that it was ‘*committed to addressing the issues identified….in order to improve the quality of care and outcomes for people with learning [intellectual] disabilities and family carers*’ (Department of Health, 2013: 2). One of the ways in which it committed to addressing the issues identified was through a mortality review process. NHS England worked with the DH, Public Health England and other partners to assess the costs and benefits of establishing a National Mortality Review Body to review deaths of people with intellectual disabilities as recommended by CIPOLD. NHS England’s Business Plan for 2013/4 to 2015/6 committed to establishing such a mortality review function and after detailed scoping work and specification development, a tender for the work was announced in 2014 and awarded to a team based at the University of Bristol in 2015. The LeDeR programme in England was commissioned by the Healthcare Quality Improvement Partnership (HQIP) on behalf of the funder, NHS England.

## The LeDeR programme in England

The initial (and continuing) aim of the LeDeR programme in England was to support improvements in the quality of health and social care service delivery for people with intellectual disabilities and to help reduce premature mortality and health inequalities in this population. This was to be achieved by supporting local agencies to review the deaths of all people with intellectual disabilities (aged 4 years and over) using a standardised review process so that recurrent themes and significant issues could be identified and addressed at local, regional and national levels. The initial terms of reference included establishing a methodology for the case review of deaths of people with intellectual disabilities; developing structures for the collection of information about deaths; supporting locally based teams to conduct reviews of deaths; and collating, analysing and reporting data submitted from reviews of deaths.

The tender for the LeDeR programme described it as a service improvement initiative, not a research programme per se. The focus was on delivering a product, the process for undertaking mortality reviews, rather than garnering longitudinal research evidence about health inequalities as experienced by people with intellectual disabilities.

## Establishing the programme

The LeDeR programme commenced in June 2015, and a number of specific ‘set up’ activities took place over succeeding months.

From the outset, two advisory groups were formed: the first was a group of people with intellectual disabilities drawn from three self-advocacy organisations and a national group representing people with intellectual disabilities; the second was a multidisciplinary group representing a range of statutory and voluntary sector agencies and family members. Each group has continued to meet twice yearly, following broadly the same agenda, with a representative from the group of people with intellectual disabilities attending the multidisciplinary group meetings to share their views. The initial membership of both groups has reduced in size over the 5-year period but has been supplemented by new additions to the groups such that overall membership has remained stable.

The inclusion criteria for the programme and the definition of ‘learning disabilities’ to be used was established early on. Initially the programme included only people with intellectual disabilities aged 4–74 years (inclusive) at the time of their death. The introduction of the national guidance on Learning from Deaths (National Quality Board, 2017) required the removal of the upper age limit and from 1st April 2017 all deaths of people with intellectual disabilities aged 4 years and above have been included, irrespective of the cause of death or place of death. The definition of ‘intellectual disabilities’ adopted was that used in national policy relating to people with ‘learning disabilities’ (Department of Health, 2001: 14).

The footprint for the LeDeR programme was the NHS regional structure, initially of four regions (subsequently increased to seven). Within each region was a number of local areas, usually delineated by Clinical Commissioning Group boundaries. Each local area, or a combination of local areas working together, was required to form a LeDeR programme steering group to guide the establishment of the programme, oversee its activities, and monitor and take forward the findings from the mortality reviews into improvements in services. Most steering groups were formed from existing multiagency networks, such as within safeguarding teams or those specifically focused on developments to services for people with intellectual disabilities. The programme was to be run on a de-centralised model: guidance was provided by the national team, but how it was enacted was interpreted locally, resulting in some variation in the programme’s implementation across the country.

The system through which deaths could be notified to the programme was subject to consultation and development, but also shaped by social factors. The key goal was to provide a secure point of contact with multiple entry points (e.g. telephone, website link, post) through which anyone (e.g. health or care professionals, people with intellectual disabilities or family members) could notify a death. A significant part of the rationale for this was the non-mandatory reporting of deaths of people with intellectual disabilities; some families were concerned that not all deaths would be notified if the process was limited to health or social care professionals only. It reflected what we interpreted as a degree of suspicion and a lack of trust by some families who were aware of reports, reviews and campaigns about contributory factors to premature deaths of people with intellectual disabilities.

Data sharing approval for the mortality reviews was sought and agreed by the NHS Health Research Authority Confidential Advisory Group (CAG) under Section 251 of the NHS Act 2006. This allows the common law duty of confidentiality to be overridden to enable disclosure of confidential patient information for specific purposes, where it is not possible to use anonymised information and where seeking consent is not practical. Despite the S251 approval being in place, several areas required local data sharing agreements to supplement this.

Attached to each local steering group were one or more local area contacts who acted as the link between the LeDeR programme team, the local steering group and local reviewers. The local area contacts received notifications of deaths, allocated them to local reviewers, provided advice and support to reviewers, monitored the progress of reviews to ensure they were completed in a timely way, and signed off completed reviews to confirm that they were comprehensive and of a consistent standard. The identification of local area contacts varied across England: some were appointed because they held a particular role or because of their level of seniority in that role; others because of their interest and expertise in the subject matter. Each local area also identified potential local reviewers, people with a professional health or social care background with experience at a senior level. They needed to have a thorough understanding of what constitutes good practice in the care of people with intellectual disabilities, the ability to talk sensitively with bereaved family members, and confidence to question and challenge professional practice when necessary. Crucially, given the context in which the LeDeR programme was being established, they were required to have senior level approval for them to incorporate conducting reviews of deaths of people with intellectual disabilities into their day-to-day work. Local reviewers were initially trained by members of the national team at 1-day training sessions held within each region. During 2018, an e-learning facility was introduced and training moved to being a combination of e-learning and locally arranged support. By this time over 1,000 reviewers had been trained in how to conduct a LeDeR review.

### Developing the mortality review process

A consultation exercise was held over a 4 month period in 2015 to gauge the views of stakeholders about the core data to be collected at the notification, initial and multi-agency reviews of a death; the criteria for deaths requiring full multi-agency review; the standards against which ‘best practice’ should be measured; and core definitions to be used (e.g. definitions of premature deaths, avoidable deaths). Over 200 responses to the consultation, incorporating a wide range of views, were received from health and care professionals, commissioners, people with intellectual disabilities and family members. Some respondents suggested the inclusion of detailed research data about the lives in general of those who had died; others proposed a short tick box form to only extract medical information about their deaths. The finally approved review process was a pragmatic compromise between what would achieve the aims of the programme while being proportionate and practical in its application. Two key issues needed to be borne in mind. First, that this was a service improvement initiative, not a research project. Information that could have been interesting to collect, e.g. the body mass index of individuals, or whether they had previously lived in a long-stay institution, was omitted if it was not felt that it would contribute to reductions in premature deaths through improved service provision. Second, the completion of reviews was required to be at local level, by local practitioners for whom this was an ‘add on’ to their existing roles, so any undue burden on the local reviewers would have to be minimised.

A secure web-based platform through which all LeDeR reviews would take place was built and tested during 2015 and 2016.

## The LeDeR programme mortality review process

**Figure 1.**
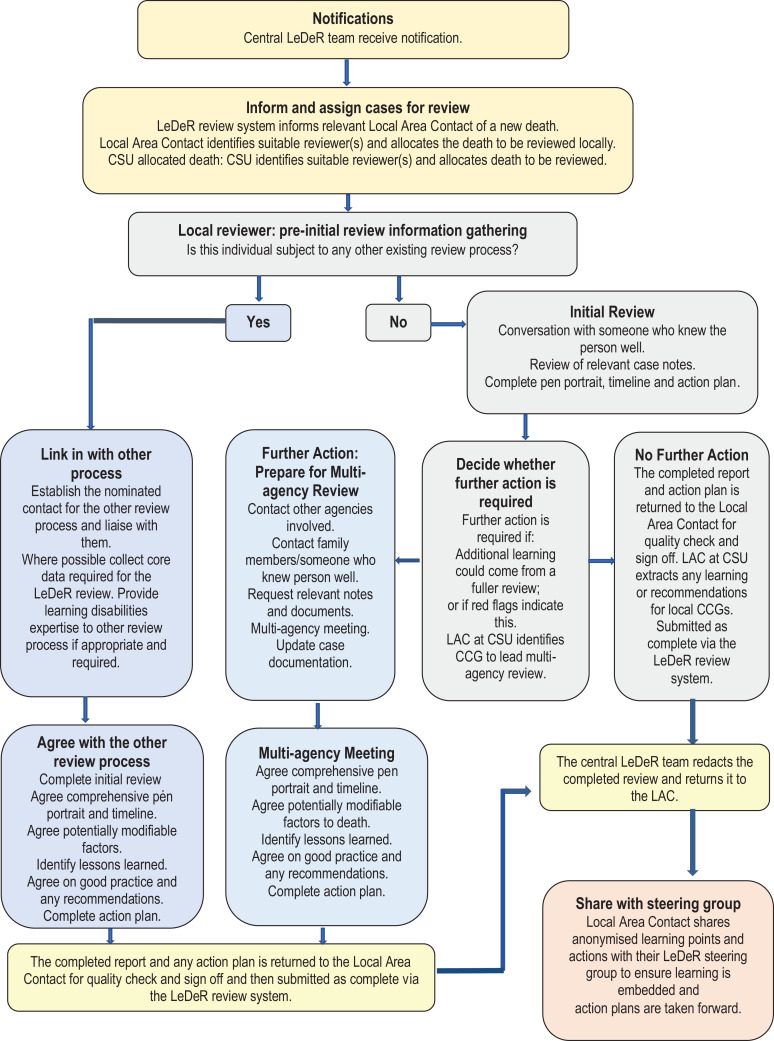
The LeDeR review process.

The LeDeR programme mortality review process is illustrated in Figure 1.

When a death is notified to the LeDeR programme, the person notifying the death is asked to provide key information about the person who has died, and for the contact details of a person who knew the deceased person well. The person’s death is logged into the LeDeR review system and the information transferred to the LeDeR local area contact in the geographical area where the deceased person had lived for allocation to a reviewer.

For each death there is an initial review. The purpose of this is to provide sufficient information to be able to determine if there are any areas of concern in relation to the care of the person who has died, or if any further learning could be gained from a multi-agency review of the death that would contribute to improving practice. The initial review involves the reviewer taking a holistic perspective of the circumstances leading to the death and not prioritising any one source of information over any other. They check and complete the information received in the notification of the death, contact a family member and/or other people who knew the deceased person well and discuss with them the circumstances leading up to the person’s death, and scrutinise at least one set of relevant care notes to extract core information about the circumstances leading to the person’s death. Based on the information obtained, the reviewer completes the standard initial review (Table 1). A draft of the initial review report may be shared with families or others to check for accuracy at any stage of the process. The completed initial review is submitted to the local area contact for quality assurance checks and ‘sign off’.

**Table 1.** The initial review.

The initial review requires the reviewer to take a holistic perspective of the circumstances leading to the person’s death which includes: A pen portrait of the person who has died. Information about the person’s health and wider support needs, and the extent to which those needs were met by health or care services. A timeline of the circumstances leading to the person’s death. The quality of care provided to the person, including any best practice that could be shared further and a grade for the quality of care on a scale of 1 (exceeded expected best practice) to 6 (care fell far short of expected good practice). Learning gained from the review, any further actions to be taken or wider recommendations made. Whether a full multi-agency review is indicated.

The purpose of the multi-agency review is to include the views of a broader range of people and agencies who have been involved in supporting the person who has died, including the family, where it is felt that further learning could be obtained from a more in-depth analysis of the circumstances leading up to the person’s death. Key indicators that a multi-agency review may be required are presented in Table 2. In summary, these are: if any ‘red flags’ were raised during the course of the review (e.g. if the person might have been subject to neglect or abuse or if their needs had not been met); if the person had been thought to have received sub-standard care that may have affected their wellbeing; if anyone had raised concerns about the person’s care; or if the reviewer felt that a fuller review of the death was required because of a lack of information or a lack of clarity about the circumstances of the person’s death.

**Table 2.** Key indicators that a multi-agency review may be required.

The initial review indicates ‘red flag’ responses – responses to standard questions that may indicate concern e.g. if the person may have been subject to abuse or neglect; there had been significant or ongoing safeguarding concerns in the previous year; there were gaps in service provision that may have contributed to the person’s death; there were delays in the person’s care or treatment that may have adversely affected their health; a Do Not Attempt Cardiopulmonary Resuscitation order was not correctly completed or followed; legislation such as the Mental Capacity Act had not been followed correctly. The assessment of the care received by the person was graded as 5 or 6: Grade 5: Care fell short of expected good practice and this significantly impacted on the person’s wellbeing and/or had the potential to contribute to the cause of death. Grade 6: Care fell far short of expected good practice and this contributed to the cause of death. There had been concerns raised about the care of the person who has died, by the person notifying the death or those contributing to the review. The reviewer felt that further learning could be gained that could contribute to improving practice.

The focus of the multi-agency review is to discuss any potentially avoidable contributory factors that had been identified relating to the person, the environment, their care and its provision, and the way services were organised and accessed. A decision is then made about whether the person’s death at that time was potentially avoidable, and the rationale provided for this. An avoidable death is one where there were aspects of care and support that, had they been identified and addressed, may have changed the outcome and on the balance of probability the person may have lived for another year or more. Finally, the multi-agency meeting identifies any lessons learned and makes recommendations for changes to local practices or wider. The reviewer provides feedback to the family if this has been agreed and submits the completed documentation to the local area contact for quality assurance checks and ‘sign off’. Once ‘signed off’ as approved by the local area contact, completed reviews are redacted by the centrally-based LeDeR team and returned to the local area contact to present to the steering group to guide the implementation of any actions. More detailed information about the LeDeR review process is available on the LeDeR website at: http://www.bristol.ac.uk/sps/leder

Essentially, mortality reviews need to be free from bias and prejudice that could arise when the reviewer, or others influencing the findings of the review, are not working from a neutral stance. The LeDeR guidance stipulates that those involved in LeDeR reviews of deaths should not have been involved in the direct care of the person who has died, and if possible, not have worked closely with those who did provide care to the person and their family. When reviewing a death, reviewers are expected to do so with impartiality – challenging the ‘status quo’ to identify system weaknesses and opportunities for learning while making decisions based on objective criteria (University of Bristol, 2019b). The extent to which local area contacts and reviewers can remain impartial is a moot one, given the apparent defensiveness of some local services in England about the quality of care provided in general (BBC News, 2018; Francis, 2013; House of Commons, 2017) and in relation to people with intellectual disabilities (Mazars, 2015). In 2018, a LeDeR review was successfully challenged by family members concerned that the local Clinical Commissioning Group had altered the findings of the review into their son’s death (BBC News, 2019, 2020) and an independent review was commissioned (NHS, 2019). The main benefits of having local area contacts and reviewers to review deaths in their own area are considered to be the ease with which the reviews could be undertaken in terms of accessing case notes and key professionals, and having a ready knowledge of systems and processes in operation. These, however, could be factors that lead to assumptions being made about deaths that may not be borne out by external scrutiny. Anecdotally, some local area contacts and reviewers have also commented that they have been surprised at what has been uncovered in reviews, that they didn’t believe that poor practice could happen to the extent that it had ‘on their patch’ and that they had strengthened their recommendations as a result. In 2019, the North of England Commissioning Service (NECS) was commissioned by NHS England to carry out some of the LeDeR reviews on behalf of CCGs and over time, a greater number of LeDeR reviews is expected to be conducted independently of local areas.

## The implementation of the mortality reviews

The first pilot site for the LeDeR programme, from January 2016, was the North East and Cumbria. The region already had an existing Intellectual Disability Network and had previously trialed some hospital-based mortality reviews of people with intellectual disabilities. Further pilot sites were introduced in 2016 to allow learning to be more evenly spread across England. In each pilot site there was an approximate 3-month preparation period, to identify key roles, train local reviewers and ensure governance systems were in place for the reviews. Support was provided by the University of Bristol LeDeR team in diverse ways, including sharing template documents (e.g. terms of reference, role descriptions), helping to problem-solve challenges (e.g. communicating to different audiences about the programme), providing advice (e.g. about information governance and data sharing), keeping in touch (e.g. by attending steering group meetings) and responding to ad hoc queries and concerns. The LeDeR team then supported each pilot site for a 4-month period as the site team started reviewing deaths. After that time, support from the University of Bristol team was phased out and NHS England became responsible for ensuring the completion of local reviews of deaths.

A significant challenge prior to more active NHS England involvement, was that the piloting of mortality reviews was being led and supported by an external agency. Greenhalgh (2018) notes the ‘subtle but profound influences of peer opinion leaders’ (p. 188) and it was not until the introduction in 2017 of regional coordinators, roles undertaken by senior staff within health and social care services, that the implementation of the LeDeR programme really stepped up a pace.

The pilot sites identified several key tasks they needed to consider and complete in relation to conducting reviews of deaths of people with intellectual disabilities. Some were relatively straightforward to address; others have remained significant challenges to the programme and are reflected on in the next section. Key issues faced by the pilot sites included:

- Estimating the expected number of deaths in each geographical area and recruiting sufficient reviewers to undertake these reviews.
- Ensuring that local and regional accountability and governance arrangements were in place for the work.
- Ensuring that practitioners were confident to share personally identifiable information under the auspices of the CAG Section 251 approval and additional data sharing agreements where required.
- Aligning the programme with other investigation and mortality reviews (e.g. safeguarding reviews, serious incident reviews, the statutory child death review process) to avoid duplication.
- Assuring the independence of reviewers from the care providers of those who had died.
- Ensuring the quality of the reviews.
- Keeping people updated about the progress of the programme while not raising expectations that deaths from outside the pilot areas would be reviewed before the wider roll out.

Learning from the pilot sites was shared at regional Learning and Sharing events in 2016 and 2017 and is summarised in Table 3.

**Table 3.** Commonly reported learning from the LeDeR programme pilot sites.

Communicate early, often and at a national and local level. Use local existing mechanisms where possible when establishing a steering group. Seek the correct membership early on, but don’t stop progress in seeking perfection. Important contributors to steering groups include primary care, coroner’s office, local authority, commissioners, providers and families. Ensure strong governance and decision making is in place at steering group level, with the consistent presence of an effective Chair and sustainable, robust governance arrangements. Sufficient, and sufficiently skilled and committed local area contacts and reviewers are crucial. The allocation of a death to review soon after a reviewer has completed their training can help reviewers to apply their training, keeps motivation levels high and contributes to reviews being completed in a timely way. It works better when local area contacts are appointed and trained before reviewers, so that they can attend and support reviewer training. The use of anonymised case studies in training helps understanding and builds commitment. Reviewers require support structures to be in place, and lines of authority and responsibility need to be carefully considered between their employer and the local area contact for the LeDeR programme. Ensure reviewers have sufficient dedicated time required to complete their reviews. It helps for local area contacts to regularly monitor the flow of reviews through the LeDeR system, and to take action if reviews appear not to be progressing. All staff involved need to be reminded of their information governance responsibilities and how these apply to the LeDeR process. All pilot sites believed that the work needs to be established on a mandatory footing. The programme has required a cultural shift for many – commonly referred to by the pilot sites as the need to ‘change hearts and minds’. Attention is needed locally and nationally about where this is most needed and how to do so effectively. A key contributor to this is the provision of evidence about the effectiveness of mortality reviews in improving health and social care services for people with learning disabilities.

The roll-out of the LeDeR programme across England took place during 2017. NHS England established a national operational steering group and each of the four NHS regions at that time recruited a regional coordinator to guide and take responsibility for the implementation and management of the LeDeR programme across each region. The readiness of local areas to start reviewing deaths differed; some, inevitably, took longer than others to establish a local steering group, identify and train reviewers, and ensure that arrangements were in place to begin reviewing deaths. There was no single ‘start’ date for the whole of England, and only when a local area was prepared in this way did they or their regional coordinator inform the University of Bristol-based LeDeR team that they could receive details about deaths of people with intellectual disabilities in their area. Deaths occurring before the local start date were not generally included in the LeDeR programme. By the end of 2017 there was a LeDeR steering group in all but one area, each local area had trained some reviewers and processes were in place throughout England to review the deaths of people with intellectual disabilities.

The programme was therefore piloted and implemented in a 2-year period. In many respects this seems a long time, especially when considering the urgent need to reduce premature death; however it also felt ‘rushed’ – some of the issues raised in the pilot sites remained outstanding (e.g. the capacity of reviewers to complete reviews in a timely way) and the pilots were not fully evaluated before proceeding to roll-out. As such, the programme was implemented across England without a full cost-benefit analysis following the pilot site involvement, and on the basis of voluntary, not mandatory participation.

## Reflections on establishing the LeDeR programme

The LeDeR programme has now been conducting reviews of the deaths of people with intellectual disabilities for more than 2 years, which has afforded an opportunity to reflect not only on how the programme was established, but also the effectiveness of some of the decisions made during that time. That the programme of reviews has continued, without it being a mandatory requirement established in law, is testament to the commitment and hard work of all of those involved and the perceived benefits of using mortality reviews as a vehicle for identifying necessary service improvements. Over 7,000 deaths have been notified to the programme from its inception to May 2020, approximately 86% of the expected number of deaths of people with learning disabilities each year. Just under half of all the deaths had been reviewed by May 2020.

### The timely completion of reviews

A key challenge that has faced the LeDeR programme since the pilot phase has been the completion of reviews in a timely way, largely driven by five key factors: (i) large numbers of deaths being notified before full capacity was in place locally to review them; (ii) a disjunct between the number of prospective reviewers attending reviewer training and the number going on to review deaths; (iii) reviewers having sufficient time away from their other duties to be able to complete a mortality review; (iv) the LeDeR mortality review process not being formally mandated; and (v) the requirement for a holistic review of the circumstances leading to the death of a person. Holistic reviews of the deaths of people with intellectual disabilities are more time-consuming than other mortality review processes which rely solely on single case note review (e.g. national mortality case record review, see Royal College of Physicians, 2016), clinician completed questionnaires (e.g. National Confidential Inquiry into Suicide and Safety in Mental Health – see University of Manchester, 2018), or confidential expert review of full medical records (e.g. Maternal, Newborn and Infant Clinical Outcome Review Programme – see Knight et al., 2018). They are important because people with intellectual disabilities more frequently have multimorbidity (two or more chronic conditions in addition to intellectual disability) than people without intellectual disabilities (Cooper et al., 2015; Hermans and Evenhuis, 2014; McCarron et al., 2013), so are often in contact with a wide range of service providers, each of whom is likely to have information pertinent to a review of the person’s death. Taking into account the views of families, or those who knew the individual best, is crucial in mortality reviews of people with intellectual disabilities; they often hold information not otherwise recorded in medical or care notes. Successive investigations have noted concerns about health and social care professionals not listening to those who knew the person well when they voiced concerns about the person’s health (Mazars, 2015; Parliamentary and Health Service Ombudsman, 2009; Heslop et al., 2013).

Attempts by NHS England to resolve these concerns included: the introduction of performance indicators to local areas; recruitment of regional coordinators to work with local steering groups; and the provision of additional funding to local areas to support their LeDeR work. In addition, (the former) NHS Sustainable Improvement reviewed the effectiveness of the guidance for conducting mortality reviews but no major changes were recommended to the way reviews are undertaken. In 2019, NHS England and NHS Improvement announced an additional £5 million to be invested by NHS England and NHS Improvement in 2019/2020 to increase the pace with which reviews are allocated and completed (NHS England and NHS Improvement, 2019b). The NHS Operational Planning and Contracting Guidance, 2019/20 (NHS England and NHS Improvement, 2019a) has stipulated the need for Clinical Commissioning Groups (CCGs) to have a robust plan in place to ensure that LeDeR reviews are undertaken within 6 months of the notification of the death to the local area, and the North of England Commissioning Support team has been contracted to support local areas with reviews. It is anticipated that by the end of 2020, all deaths reported to the programme will be reviewed within a 6-month timeframe.

### Inclusivity

Initially, the LeDeR programme only reviewed the deaths of people with intellectual disabilities aged 4–74 years. The lower age limit was set because before the age of 4 it can be difficult to identify that a child has intellectual disabilities unless they have a specific syndrome associated with having intellectual disabilities. The upper age limit always sat a little uncomfortably and was based on definitions of ‘premature deaths’ described in the mortality profiles by Public Health England (Public Health England, 2019). To come in line with the National Guidance on Learning from Deaths (National Quality Board, 2017) introduced in England in 2017, the upper age limit was removed, and the LeDeR programme subsequently reviewed all deaths of people with intellectual disabilities aged 4 years and over. A more inclusive approach from the outset would, in hindsight, have been helpful, because of the ease with which age-related data prior to and after April 2017 could be compared, and because scrutiny of service provision for the oldest people with intellectual disabilities can offer useful insights into how it could be improved (Bigby, 2005; David et al., 2015; Lin et al., 2011).

### The extent of reviewing deaths in a population

This issue of whether all deaths (within the eligibility criteria), or just a proportion, should be reviewed, has been raised repeatedly since the inception of LeDeR, and more frequently as the challenge of completing reviews in a timely manner has persisted. The National Guidance for Learning from Deaths in England (National Quality Board, 2017) obliges NHS Trusts to review the deaths of all people with intellectual disabilities, a requirement guided in part by the findings of CIPOLD (Heslop et al., 2013) which noted that some deaths had not been referred for external scrutiny by coroners or internal review (by safeguarding boards or serious incident review processes) when this should have been the case. The continuing need to review all deaths has since been supported by the early findings of the LeDeR programme, which reported an apparent under-representation of deaths of people with intellectual disabilities reported to a coroner, continuing inequities in access to health and care services, examples of diagnostic overshadowing, and that fewer than half (48%) of the deaths reviewed in 2018 received care that the reviewer felt met or exceeded good practice (University of Bristol, 2019a). In addition, although the findings and resulting actions from individual reviews of deaths can be vital levers for service improvement, it is the collective experiences of people with intellectual disabilities that provide a strong evidence-base for change, and the two need to go hand-in-hand. Using evidence solely from collated reports of deaths, there is a danger of people assuming that ‘it doesn’t happen in my service/geographical area’; using evidence solely from ad hoc individual reviews runs the risk of assuming that problems with care are isolated incidents that could not be repeated. In our view, until we have clear evidence that the inequities experienced by people with intellectual disabilities have reduced and the reduction has been maintained, we should continue to cast a spotlight on the deaths of all people with intellectual disabilities.

### The independence of the programme

The perceived independence of the LeDeR programme is an issue on which it is also helpful to reflect. At national level, the LeDeR programme was established in such a way that the roles of the University of Bristol, NHS England and local areas have, to a large extent, been separate but inter-dependent. The University of Bristol-based LeDeR team undertook the initial set-up activities for the programme; established the central notification system for reporting deaths of people with intellectual disabilities; training for reviewers and local area contacts until September 2018; quality assurance of completed reviews until November 2018; reporting on the progress of reviews; and the coding, collation and dissemination of information from completed reviews. Thus, the University of Bristol team provided the infrastructure to enable mortality reviews to take place. NHS England have overall responsibility for ensuring the completion of the reviews by local systems, and assuring that local systems take forward the findings from reviews into local and service improvements. NHS England also have a responsibility to ensure that any national recommendations relevant to their remit are taken forward. Local areas provided governance arrangements for the programme locally, implement the programme at local level, and make local service improvements based on the learning from reviews.

This was reinforced in 2019 when NHS England included in the NHS Operational Planning and Contracting Guidance, 2019/20 the requirement for CCGs to be members of LeDeR steering groups and have a named person with lead responsibility for LeDeR (NHS England and NHS Improvement, 2019a). It also required CCGs to have systems in place to analyse and address the themes and recommendations from completed LeDeR reviews, and to submit an annual report demonstrating local action taken and outcomes from LeDeR reviews. This division of responsibilities and its complexity has, at times, created confusion and challenges as it has evolved, but the successful interface between all parties has been essential for the development and continuation of LeDeR. Where the independence of the programme nationally is particularly important, is in the accurate and unbiased reporting of findings from collated mortality reviews that is free from external influence.

Without a nationally mandated process for undertaking reviews of the deaths of people with intellectual disabilities established in law, there has been, inevitably, some variation in the local delivery of the LeDeR programme. This, alongside a shortage of reviewers and the challenges of completing reviews in a timely way, has resulted in difficulties in allocating some deaths to reviewers wholly independent of direct care provider agencies in some areas. In addition, some have concerns that the recent requirements for commissioners of services to be more closely involved with the LeDeR programme at local level (NHS England and NHS Improvement, 2019a), may potentially risk further challenges to the independence of mortality reviews. Work considering the future approach to LeDeR reviews will need to consider this. A strategy to mitigate this could be the introduction of an independent, national overview panel, as recommended by CIPOLD (Heslop et al., 2013) to consider how the reviews have been carried out, the quality of a random sample of completed reviews, and act as an arbiter in case of disputes.

### The effectiveness of the programme

There are several measures of the effectiveness of a mortality review programme, such as the number of deaths reviewed, or the timeliness within which reviews are completed, as already alluded to. But there is little point in reviewing deaths if the lessons learned and recommendations made are not acted upon to prevent other deaths from similar circumstances. For its ongoing success, the LeDeR programme must lead to better outcomes for people with intellectual disabilities. To be assured of this, we need to examine not only what has happened to people leading up to their deaths, but also to any learning and recommendations that are made as a result and to the effectiveness or otherwise of their enactment.

Individual level reviews provide a complementary perspective to population level trends; together their findings can form an important component of planning and allocating health resources and implementing health and care improvement strategies. The LeDeR programme has not yet been able to fully assess the impact of the programme of reviews or the recommendations made at individual or population level. At present, completed reviews of deaths document any learning that has been gained from the review, and may make recommendations for future preventative action; these are included in the analysis of completed reviews undertaken by the University of Bristol team and a summary published in the LeDeR annual reports. The enactment of the recommendations is dependent upon CCGs and providers taking action locally in the way services are delivered. While some information is collected centrally by NHS England, there have been some challenges to the NHS in systematically collating in a structured way the outcomes of all local recommendations and reporting these nationally.

At a national level, the University of Bristol team, in conjunction with people with intellectual disabilities and their families, and a range of representatives from professional and practitioner agencies, identify several key recommendations from the analyses of collated reviews of deaths. These are published in the LeDeR programme annual reports. Alongside the publication of the LeDeR annual report, NHS England publishes an ‘Action from Learning’ report, which includes an update about actions that have been taken in relation to these recommendations at national and local levels. In 2019, the Action from Learning report included commentary about the early identification of a deterioration in the physical health of people with intellectual disabilities; early recognition and treatment of sepsis; raising awareness about constipation and dysphagia; and improving the understanding of, and adherence to, the Mental Capacity Act (NHS England and NHS Improvement, 2019b).

There currently appears to be no specific mechanism through which the findings and recommendations of the six national HQIP-enabled Clinical Outcome Review programmes in England are collated and considered together, although some of the recommendations may share commonalities and overlaps. There is a pressing need for a national mortality oversight body to draw together the findings and recommendations across the different mortality review programmes, prioritise recommendations, and oversee their implementation. In addition, the involvement of implementation scientists is required to consider the complex systems into which recommendations are made and improve the effectiveness of the resulting service improvements.

## Conclusions

The LeDeR programme in England is the first of its kind in the world. Commencing in 2015, it has been implemented at a time of considerable concern about premature deaths of people with intellectual disabilities, and the introduction of the NHS-wide Learning from Deaths Framework in 2017. The LeDeR programme now reviews the deaths of all people with intellectual disabilities aged 4 years and over, with the aim of supporting improvements in the quality of health and social care service delivery and helping to reduce premature mortality and health inequalities in this population. It has taken time to embed the LeDeR programme into existing systems and processes, and its lack of a mandatory footing has arguably led to a vulnerability to variation in local delivery and of other priorities contributing to delays in the timeliness with which reviews of deaths are completed. That so many reviews have been completed to a high standard, with evidence of insightful and reflective practice and clear recommendations for improvements in service delivery, is heartening. So too are the reviews that indicate exemplary care that could and should be shared for wider benefit. Evidence from mortality reviews, however, is important but not sufficient. The focus of the LeDeR programme now needs to be on taking forward the findings of mortality reviews into service improvements at local, regional and national levels. A coordinated approach is required to draw together the findings and recommendations, identify activities being taken locally and their effectiveness, prioritise national recommendations and oversee their implementation. It is important too to consider the findings of the LeDeR programme with those of the other national Clinical Outcome Review programmes. It is such actions that will help achieve real progress for people with intellectual disabilities and give assurance that the LeDeR programme has achieved its aims of supporting improvements in the quality of health and social care service delivery for people with intellectual disabilities and helping to reduce premature mortality and health inequalities in this population.
